# Both sedentary time and physical activity are associated with cardiometabolic health in overweight adults in a 1 month accelerometer measurement

**DOI:** 10.1038/s41598-020-77637-3

**Published:** 2020-11-25

**Authors:** Tanja Sjöros, Henri Vähä-Ypyä, Saara Laine, Taru Garthwaite, Minna Lahesmaa, Sanna M. Laurila, Aino Latva-Rasku, Anna Savolainen, Annika Miikkulainen, Eliisa Löyttyniemi, Harri Sievänen, Kari K. Kalliokoski, Juhani Knuuti, Tommi Vasankari, Ilkka H. A. Heinonen

**Affiliations:** 1 Turku PET Centre, University of Turku and Turku University Hospital, P.O. box 52, 20521 Turku, Finland; 2 The UKK-Institute, Tampere, Finland; 3 Turku Heart Center, Turku University Hospital, Turku, Finland; 4 Department of Biostatistics, University of Turku, Turku, Finland; 5 Rydberg Laboratory of Applied Sciences, University of Halmstad, Halmstad, Sweden

**Keywords:** Metabolic diseases, Dyslipidaemias, Metabolic syndrome, Obesity, Pre-diabetes, Atherosclerosis

## Abstract

The aim of this study was to examine the associations of cardiometabolic health markers with device-measured sedentary behavior (SB) duration and different intensities of physical activity (PA) among overweight working-aged adults with low self-reported PA levels. This cross-sectional analysis included 144 subjects (42 men) with mean age of 57 (SD 6.5) years and mean BMI of 31.7 (SD 4) kg/m^2^. SB and standing time, breaks in sedentary time, light PA (LPA) and moderate-to-vigorous PA (MVPA) were measured for 4 consecutive weeks (mean 25 days, SD 4) with hip-worn accelerometers. Fasting plasma glucose, insulin, HbA_1c_, triglycerides and total cholesterol, HDL and LDL were measured from venous blood samples. HOMA-IR index was calculated as a surrogate of insulin resistance. The associations were examined using linear models. LPA, MVPA, and daily steps associated with better insulin sensitivity and favorable plasma lipid profile, when adjusted for age, sex and BMI, whereas greater proportion of SB associated with insulin resistance and unfavorable lipid profile. As all PA intensities associated with better cardiometabolic health, the total daily duration of PA may be more relevant than intensity in maintaining metabolic health in overweight adults, if the current guidelines for PA are not met.

**Trial Registration:** ClinicalTrials.gov NCT03101228, registered 05/04/2017, https://clinicaltrials.gov/show/NCT03101228.

## Introduction

Insufficient amount of moderate-to-vigorous physical activity (MVPA) and prolonged periods spent sedentary (~ sitting) have been identified as important predictors of metabolic disorders, type 2 diabetes, cardiovascular disease (CVD) and premature mortality^1–6^. Unfortunately, the majority of the world’s population do not meet the current guidelines for MVPA^7^.

About 12% of the adult population of the world is obese [body mass index (BMI) ≥ 30 kg/m^2^], and obesity-related diseases caused about 4 million deaths per year in 2015^8^. Moreover, overweight is a risk factor for developing the metabolic syndrome (MetS) and cardiometabolic diseases later in life, even in apparently healthy overweight adults^9^.

Recently, there has been a growing interest in objectively measuring sedentary behavior (SB) instead of relying on self-reports, to understand its associations and potential in the prevention of obesity and metabolic diseases as well as premature mortality^10^. In cross-sectional settings, greater volume of device-measured SB time has been associated with higher plasma glucose, insulin and triglyceride levels as well as lower levels of advantageous HDL-cholesterol and larger waist circumference^11^. Additionally, device-measured SB time is associated with glucose intolerance and an increased risk of MetS and type 2 diabetes^4,12–16^. Whereas in a population-based sample of English adults MVPA associated with BMI, waist circumference, HbA_1c_ and HDL, but sedentary status associated solely with HDL^17^.

Breaking up SB by sit-to-stand transitions has been associated with smaller waist circumference^18^. Moreover, in short-term cross-over interventions, active interruptions to sitting by physical activity (PA) breaks have led to attenuated postprandial glucose- and insulin responses^19^. However, in some studies, breaks in sedentary time have not been identified with beneficial associations^4^. Thus the evidence on the beneficial effects of interrupting sitting with activity breaks remains somewhat controversial.

On the other hand, the objective methods to measure SB are not without limitations. One issue to consider is the coverage of the measurement. The measurements of PA and SB with for example accelerometers have thus far generally lasted for up to 7 days. This may be a weakness in the current evidence as it is possible that wearing a measurement device actually has an impact on one’s sitting and PA behaviors, at least in short measurement periods.

There appears to occur considerable intra-individual variation in daily PA and SB time. Measured by a pedometer, at least 30 consecutive days of measurement are needed to reach mean absolute percentage error lower than 10% or at least 14 consecutive days to reach intraclass correlation coefficient (ICC) of ≥ 0.9, compared to full 365 days of monitoring^20^. Therefore, in this study, we aimed at measuring PA and SB, but also the amount of standing for four consecutive weeks. Furthermore, another issue in device-measured determination of PA is the temporal resolution of data collection, and capability to directly separate different behaviours, such as sitting and standing, instead of using counts. Consequently, in the present study all activities were measured with tri-axial accelerometers worn on the hip, in 6 s intervals, for the whole 4-week period, to obtain the best possible precision to describe individual SB and PA levels.

As increased fasting insulin and glucose levels are well-established indicators of insulin resistance in population studies, the purpose of this study was to examine the associations between plasma insulin and other markers of metabolic health with device-measured SB and standing time, breaks in sedentary time, light physical activity (LPA) and MVPA in an overweight working aged population with low self-reported PA levels. Our particular interest was to investigate whether our device-measured physical (in)activity markers associate with insulin and glucose concentrations after adjustment for common confounding risk factors such as age and BMI; and which SB or PA categories associate the strongest with the variables of interest.

## Methods

This study was a one-arm explorative observational study consisted of the screening phase of an intervention. The study was conducted at the Turku PET Centre, Turku, Finland between April 2017 and May 2019. This study was conducted according to good clinical practice and the Declaration of Helsinki. Informed consent was obtained from the subjects before entering the study. The study was approved by the Ethics Committee of the Hospital District of Southwestern Finland (16/1810/2017). The study is registered at ClinicalTrials.gov (NCT03101228, 05/04/2017).

### Subjects

The participants in this study were recruited from the local community by newspaper advertisements and bulletin leaflets. Criteria for selecting the subjects were as follows: Age 40–65 years, BMI 25–40 kg/m^2^, and according to self-reports, the subjects should not meet the current recommendations for physical activity and they should sit a major proportion of the day. The exclusion criteria were as follows: history of a cardiac event, insulin or medically treated diabetes, abundant use of alcohol (according to national guidelines), use of narcotics, smoking of tobacco or consuming of snuff tobacco, inability to understand written Finnish and any chronic disease or condition that could create a hazard to the subject safety or endanger the study procedures.

### Study design

The eligible volunteers were interviewed, and during the interview, BMI, waist circumference and blood pressure were measured. The subjects received an accelerometer, which they were instructed to wear on the right hip for four consecutive weeks, starting the following morning. They were instructed to wear the accelerometer during waking hours, except for activities where the devise would be exposed to water. Moreover, they were advised to maintain their habitual activities and ways of life during the measurement. During the 4-week measurement, at their most convenient time, they were instructed to visit the laboratory for fasting venous blood samples.

### Measurements

SB and PA were measured for four weeks with hip-worn tri-axial accelerometers (UKK AM30, UKK-Institute, Tampere, Finland) using a digital triaxial acceleration sensor (ADXL345; Analog Devices, Norwood MA, USA), that was attached to a flexible belt. The accelerometer stored the acceleration signal with 100 Hz sampling frequency, ± 16 *g* measurement range, and 4 m*g* (milligravity) measurement resolution. After the measurement, the collected acceleration data were transferred to hard disk for further analysis. The analysis was performed with Microsoft Excel 2010 Visual Basic for Applications (VBA) program (Microsoft Corporation, Santa Rosa, CA, USA).

The collected accelerometer data was analyzed in six-second epochs using validated mean amplitude deviation (MAD) and the epoch-wise MAD values were converted to metabolic equivalents (MET)s (3.5 ml/kg/min of oxygen consumption)^21^. LPA was defined as MET value higher than or equal to 1.5 and less than 3.0 (MAD value between 22.5 and 91.5 m*g*), MVPA as MET value higher than or equal to 3.0 (MAD over 91.5 m*g*) and vigorous PA as MET value higher than or equal to 6.0 (MAD over 414.5 m*g*). However, vigorous PA was added to moderate-intensity PA and presented as MVPA, because the data was non-normally distributed and could not be improved with any data transformations. Additionally, the epoch-wise MET values were smoothed with 1-min exponential moving average and the daily peak 1-min exponential moving average of epoch-wise MET values was determined. The daily mean MET value was calculated from epoch-wise MET values.

The body posture was determined with angle for posture estimation (APE) method only for the epochs, which had MAD value lower than 22.5 m*g*^22^. The classification of body posture was determined by two assumptions; the earth’s gravity vector is constant and the body’s posture while walking is upright. During walking, the accelerometer orientation in terms of the gravity vector was taken as the reference vector. The posture was determined from the incident accelerometer orientation in relation to the reference vector. The epochs having APE values less than 11.6° were classified as standing and epochs having APE value at least 11.6° as SB. In standardized conditions, SB can be separated from standing with 95% accuracy. In free-living conditions, the agreement between the posture classification from simultaneous thigh-worn and hip-worn data has been about 90%^22^. The daily number of breaks in sedentary time denoted the number of SB periods during which the 1-min exponential moving average of the estimated MET value was less than 1.5 and which ended-up with a clear vertical acceleration and subsequent standing position or movement^22^.

The step detection algorithm splits the measured acceleration into vertical and horizontal components. The vertical component is band-pass filtered (1–4 Hz) and positive values are integrated. When the integral value exceeds the specified limit, the step is detected. The step algorithm requires about 3 km/h walking speed to detect every step^22^.

A period was classified as a non-wear time, if the acceleration of each three-measurement axis remained within 187.5 m*g* range for at least for 30 min time. Wear time of 10–19 h/day and 4 days of measurement were considered valid. Daily measurement time exceeding 19 h indicates that a participant has likely slept with the accelerometer, and therefore measurement hours exceeding 19 h/day were subtracted from the SB time. Additionally, proportions of different activity intensities per day were calculated, and presented as percentage of wear time.

Venous blood samples were draw after at least 10 h of fasting and analyzed at the Turku University Hospital Laboratory. Plasma insulin was determined by electrochemiluminescence immunoassay (Cobas 8000 e801, Roche Diagnostics GmbH, Mannheim, Germany). Plasma glucose was determined by enzymatic reference method with hexokinase GLUC3; and plasma triglycerides, total, LDL and HDL cholesterol by enzymatic colorimetric tests (Cobas 8000 c702, Roche Diagnostics GmbH, Mannheim, Germany). HbA_1c_ was determined by turbidimetric inhibition immunoassay (Cobas 6000 c501, Roche Diagnostics GmbH, Mannheim, Germany). HOMA-IR index was calculated by formula: glucose × insulin/22.5^23^.

Blood pressure was measured by a digital blood pressure monitor (Apteq AE701f, Rossmax International LtD, Taipei, Taiwan) in a seated position after at least 10 min on sitting. The mean of 2–3 measurements was used as the outcome measure. Body mass was measured by scales (Seca 797, Vogel & Halke, Hamburg, Germany) in light clothing. Body height was measured barefooted with a wall-mounted stadiometer. Waist circumference was measured with a flexible measuring tape midline between the iliac crest and the lowest rib, repeated twice or until the same measure was obtained twice.

### Statistical methods

In our sample, there was a significant difference in the fasting insulin levels between men and women. Therefore sex was included as a variable in all the analyses. The associations were examined by linear models with one categorical (sex) and three continuous variables (age, the outcome (metabolic marker) and activity measure) for BMI, waist circumference, glucose, HbA_1c_, insulin and HOMA-IR. For lipid-related outcomes, the possible use of cholesterol-lowering medication (statins) was added to the model; and for blood pressure-related outcomes, the possible presence of medical treatment of hypertension was added to the model. Additionally, further analyses with fourth continuous variable BMI were conducted to adjust for confounding overweight. Logarithmic (log10) transformations were performed when necessary to achieve normal distribution of the data. The normal distributions of the residuals were examined visually, and sensitivity analyses were performed by leave-one-out method to assure the robustness of the findings. Missing data was handled by pairwise deletion and maximum likelihood estimation. If not otherwise stated, data are expressed as means (SD). The level of statistical significance was set at 5%. All analyses were carried out with the JMP pro 13.1 for Windows (SAS Institute Inc., Cary, NC, USA).

## Results

In total, 263 subjects volunteered, of whom 102 women and 42 men were found eligible and completed the accelerometer measurements. The mean accelerometer wear time was 14.37 (SD 1.04) h/day and the mean duration of the measurement was 25 (SD 4) days. 99% of the subjects wore the accelerometer for more than 12 h/day in average and proportions of subjects with mean wear time for over 14 and 16 h were 63% and 6%, respectively. There were differences in SB time, SB percentage and standing time between men and women, but not in daily steps or PA (Table 1). The subjects spent 67.0 (SD 8.3) % of the accelerometer wear time in sedentary activities, and took in average 5265 (SD 2113) steps per day. Blood samples were collected from 102 women and 40 men, only two subjects failed to visit the laboratory.

**Table 1:** Characteristics of the study subjects by sex.

	Men	Women
n (% of total)	42 (29)	102 (71)
Age, years	58.0 (6.0)	56.4 (6.7)
Height, cm	178.8 (7.1)***	165.2 (6.1)***
Body mass, kg	101.8 (14.7)***	86.7 (13.4)***
BMI, kg/m^2^	31.8 (3.6)	31.7 (4.2)
Waist circumference, cm	116.3 (11.0)***	106.7 (10.4)***
SBP, mmHg	149 (19)	147 (20)
DBP, mmHg	91 (11)	90 (12)
HR, bpm	70 (11)	71 (11)
BPL medication, n (%)	23 (55)*	34 (33)*
CL medication (statins), n (%)	8 (11)	11 (11)
f-Glucose, mmol/l	5.9 (0.7)	5.8 (0.9)
f-Insulin, µmol/l	16.0 (10.4)*	11.8 (7.3)*
HOMA-IR	4.2 (3.0)*	3.2 (2.4)*
Triglycerides, mmol/l	1.6 (0.9)	1.4 (0.8)
Cholesterol, mmol/l	5.0 (0.7)**	5.4 (0.9)**
HDL-cholesterol, mmol/l	1.28 (0.32)***	1.66 (0.43)***
LDL-cholesterol, mmol/l	3.4 (0.7)	3.4 (0.8)
HbA_1c_, mmol/mol	37.5 (5.3)	36.9 (6.1)
HbA_1c_, %	5.58 (0.48)	5.52 (0.56)
Accelerometry, days	24 (5)	26 (4)
Wear time, h/day	14.27 (1.14)	14.41 (1.00)
Sedentary time, h/day	10.13 (1.24)**	9.42 (1.31)**
Standing, h/day	1.44 (0.44)***	2.18 (0.76)***
LPA, h/day	1.67 (0.61)	1.83 (0.45)
MVPA, h/day	1.03 (0.43)	0.98 (0.36)
PA, h/day	2.70 (0.92)	2.81 (0.70)
Sedentary proportion, %/day	71.0 (7.3)***	65.4 (8.1)***
Standing proportion, %/day	10.1 (2.9)***	15.0 (5.0)***
LPA proportion, %/day	11.7 (3.9)	12.8 (3.1)
MVPA proportion, %/day	7.3 (2.9)	6.8 (2.5)
PA proportion, %/day	19.0 (5.8)	19.6 (4.8)
Breaks in sedentary time/day	26 (7)**	30 (8)**
Daily steps	5408 (2288)	5206 (2046)
MET peak/day	4.9 (0.6)	4.8 (0.7)
MET mean/day	1.3 (0.1)	1.3 (0.1)

If not otherwise stated, the results are reported as mean (SD).

*BMI* body mass index, *SBP* systolic blood pressure, *DBP* diastolic blood pressure, *HR* heart rate, *BPL* blood pressure lowering, *CL* cholesterol lowering, *LPA* light physical activity, *MVPA* moderate to vigorous physical activity, *PA* physical activity (LPA and MVPA together), *MET* the metabolic equivalent.

Sex difference in t-test (or Fisher’s exact test, when applicable), *p < 0.05, **p < 0.01, ***p < 0.001.

### Anthropometric measures

BMI associated negatively with MVPA and especially with daily steps, breaks in sedentary time and MET peak (the mean of the daily MET peak values), when adjusted for age and sex (Table 2). BMI associated positively with SB percentage (of total accelerometer wear time) but not with total SB time. Waist circumference associated strongly with breaks in sedentary time, daily steps, MET peak, SB percentage and also standing time, whereas with LPA there was no association (Supplementary Table S1). See Supplementary Tables S2 and S3 for details of the full models.

**Table 2:** Associations between BMI and accelerometer measures, least squares effect tests with sex and age included in the model.

	*df*	F ratio	t ratio	*p*	r^2^
Sedentary %	1	7.20	2.68	0.0082	0.05
Sedentary time	1	2.27	1.51	0.13	0.02
Standing %	1	5.91	− 2.43	0.016	0.05
Standing time	1	6.73	− 2.59	0.011	0.05
LPA %	1	0.93	− 0.97	0.34	0.01
LPA time	1	1.61	− 1.27	0.21	0.02
MVPA %	1	6.92	− 2.63	0.0095	0.05
MVPA time	1	8.08	− 2.84	0.0051	0.06
PA %	1	3.79	− 1.95	0.054	0.03
PA time	1	4.88	− 2.21	0.029	0.04
Steps/day	1	13.81	− 3.72	0.0003	0.10
Breaks in sedentary time	1	16.24	− 4.03	< 0.0001	0.11
MET mean/day	1	9.08	− 3.01	0.0031	0.07
MET peak/day	1	12.63	− 3.55	0.0005	0.09

*LPA* light physical activity, *MVPA* moderate to vigorous physical activity, *PA* physical activity (LPA and MVPA together), *MET* the metabolic equivalent.

### Insulin and HOMA-IR

Fasting insulin and HOMA-IR associated negatively with PA, daily steps, breaks in sedentary time and daily MET peak and mean values, when adjusted for age and sex (Table 3, Supplementary Table S4). Greater SB percentage was associated with higher fasting insulin and HOMA-IR. The strongest associations to fasting insulin and HOMA-IR were found with daily steps and MET mean (mean of MET mean values of each day). See Supplementary Tables S5 and S6 for details of the full models. The unadjusted associations between fasting plasma insulin and accelerometer measures are presented in Supplementary Fig. S1.

**Table 3:** Associations between fasting insulin (log10) and accelerometer measures, least squares effect tests with sex and age included in the model.

	*df*	F ratio	t ratio	*p*	r^2^
Sedentary %	1	11.15	3.34	0.0011	0.12
Sedentary time	1	3.25	1.80	0.073	0.07
Standing %	1	4.24	− 2.06	0.041	0.08
Standing time	1	5.05	− 2.25	0.026	0.09
LPA %	1	5.10	− 2.26	0.026	0.09
LPA time	1	6.13	− 2.48	0.015	0.09
MVPA %	1	12.16	− 3.49	0.0007	0.13
MVPA time	1	14.03	− 3.75	0.0003	0.14
PA %	1	10.56	− 3.25	0.0015	0.12
PA time	1	12.05	− 3.47	0.0007	0.13
Steps/day	1	19.41	− 4.41	< 0.0001	0.17
Breaks in sedentary time	1	12.70	− 3.56	0.0005	0.13
MET mean/day	1	15.43	− 3.93	0.0001	0.15
MET peak/day	1	13.53	− 3.68	0.0003	0.14

*LPA* light physical activity, *MVPA* moderate to vigorous physical activity, *PA* physical activity (LPA and MVPA together), *MET* the metabolic equivalent.

### Glucose and HbA_1c_

Fasting glucose associated positively with SB percentage and negatively with standing time, MVPA, daily steps, breaks in sedentary time and MET mean and peak values, when adjusted for age and sex (Table 4). See Supplementary Table S7 for details of the full models. However, in sensitivity analyses after excluding one subject with extreme glucose value, only the associations of glucose with standing, MVPA and daily steps remained significant (p = 0.031, 0.028 and 0.0089, respectively).

**Table 4:** Associations between fasting plasma glucose and accelerometer measures, least squares effect tests with sex and age included in the model.

	*df*	F ratio	t ratio	*p*	r^2^
Sedentary %	1	5.82	2.41	0.017	0.04
Sedentary time	1	1.87	1.37	0.17	0.01
Standing %	1	4.38	− 2.09	0.038	0.03
Standing time	1	5.37	− 2.32	0.022	0.04
LPA %	1	0.92	− 0.96	0.34	0.01
LPA time	1	1.41	− 1.19	0.24	0.01
MVPA %	1	5.79	− 2.41	0.018	0.04
MVPA time	1	7.45	− 2.73	0.0072	0.05
PA %	1	3.33	− 1.82	0.070	0.02
PA time	1	4.41	− 2.10	0.038	0.03
Steps/day	1	9.54	− 3.09	0.0024	0.07
Breaks in sedentary time	1	4.98	− 2.23	0.027	0.04
MET mean/day	1	5.54	− 2.35	0.020	0.04
MET peak/day	1	4.54	− 2.13	0.035	0.03

*LPA* light physical activity, *MVPA* moderate to vigorous physical activity, *PA* physical activity (LPA and MVPA together), *MET* the metabolic equivalent.

HbA_1c_ associated negatively with breaks in sedentary time when adjusted for age and sex, but not with any other accelerometer measures (Supplementary Table S8). In sensitivity analyses, after excluding one subject with extreme HbA_1c_ value, this association turned non-significant (p = 0.089). The unadjusted associations between fasting plasma glucose and HbA_1c_ and accelerometer measures are presented in Supplementary Fig. S1.

### Plasma lipids

Fasting plasma triglycerides associated positively with SB percentage and negatively with PA, when adjusted for age, sex and usage of cholesterol lowering medication (Table 5). The associations of triglycerides with total PA and LPA were stronger than associations between triglycerides and MVPA.

**Table 5:** Associations between fasting plasma triglycerides (log10) and accelerometer measures, least squares effect tests with sex, age and cholesterol lowering medication included in the model.

	*df*	F ratio	t ratio	*p*	r^2^
Sedentary %	1	11.05	3.32	0.0011	0.09
Sedentary time	1	2.30	1.52	0.13	0.04
Standing %	1	4.69	− 2.16	0.032	0.05
Standing time	1	5.48	− 2.34	0.021	0.06
LPA %	1	7.83	− 2.80	0.0059	0.07
LPA time	1	9.20	− 3.03	0.0029	0.08
MVPA %	1	6.11	− 2.47	0.015	0.06
MVPA time	1	7.23	− 2.69	0.0080	0.07
PA %	1	9.73	− 3.12	0.0022	0.09
PA time	1	11.17	− 3.34	0.0011	0.09
Steps/day	1	7.34	− 2.71	0.0076	0.07
Breaks in sedentary time	1	7.25	− 2.69	0.0080	0.07
MET mean/day	1	8.96	− 2.99	0.0033	0.08
MET peak/day	1	3.98	− 2.00	0.048	0.05

*LPA* light physical activity, *MVPA* moderate to vigorous physical activity, *PA* physical activity (LPA and MVPA together), *MET* the metabolic equivalent.

HDL-cholesterol associated negatively with SB time and SB percentage, when adjusted for age, sex and usage of cholesterol lowering medication (Table 6). Longer duration of LPA, MVPA and total PA were associated with higher HDL. There was no association between standing and HDL. See Supplementary Tables S9 and S10 for details of the full models.

**Table 6:** Associations between plasma HDL-Cholesterol and accelerometer measures, least squares effect tests with sex, age and cholesterol lowering medication included in the model.

	*df*	F ratio	t ratio	*p*	r^2^
Sedentary %	1	9.60	− 3.10	0.0024	0.25
Sedentary time	1	5.92	− 2.43	0.016	0.23
Standing %	1	2.10	1.45	0.15	0.21
Standing time	1	2.65	1.63	0.11	0.21
LPA %	1	9.93	3.15	0.0020	0.25
LPA time	1	10.53	3.24	0.0015	0.25
MVPA %	1	7.12	2.67	0.0085	0.23
MVPA time	1	8.03	2.83	0.0053	0.24
PA %	1	11.98	3.46	0.0007	0.26
PA time	1	12.67	3.56	0.0005	0.26
Steps/day	1	9.05	3.01	0.0031	0.24
Breaks in sedentary time	1	5.56	2.36	0.020	0.23
MET mean/day	1	11.76	3.43	0.0008	0.26
MET peak/day	1	4.19	2.05	0.043	0.22

*LPA* light physical activity, *MVPA* moderate to vigorous physical activity, *PA* physical activity (LPA and MVPA together), *MET* the metabolic equivalent.

No associations were found between total or LDL-cholesterol and accelerometer measures when adjusted for age, sex and usage of cholesterol lowering medication (Supplementary Tables S11, S12). See Supplementary Table S13 for details of the full models. The unadjusted associations between plasma lipids and accelerometer measures are presented in Supplementary Fig. S2.

### Blood pressure and heart rate

No associations were found between accelerometer measures and blood pressure, even if adjusted for age, sex and usage of antihypertensive medication (data not shown). Lower resting heart rate associated significantly with more breaks in sedentary time (p = 0.018).

All the above-mentioned associations are summarized in Table 7.

**Table 7:** Summary table, associations between accelerometer measures and metabolic outcomes derived from clinical investigation and fasting blood samples measured by linear models *with age and sex included in the analyses*.

	BMI	Waist	Insulin	HOMA-IR	Glucose	HbA_1c_	Triglycerides	HDL	LDL	Cholesterol	RHR	SBP	DBP
Sedentary %	↑	↑	↑	↑	↑	ns	↑	↓	ns	ns	ns	ns	ns
Sedentary time	ns	↑	ns	ns	ns	ns	ns	↓	ns	ns	ns	ns	ns
Standing %	↓	↓	↓	↓	↓	ns	↓	ns	ns	ns	ns	ns	ns
Standing time	↓	↓	↓	↓	↓	ns	↓	ns	ns	ns	ns	ns	ns
LPA %	ns	ns	↓	↓	ns	ns	↓	↑	ns	ns	ns	ns	ns
LPA time	ns	ns	↓	↓	ns	ns	↓	↑	ns	ns	ns	ns	ns
MVPA %	↓	↓	↓	↓	↓	ns	↓	↑	ns	ns	ns	ns	ns
MVPA time	↓	↓	↓	↓	↓	ns	↓	↑	ns	ns	ns	ns	ns
PA %	ns	↓	↓	↓	ns	ns	↓	↑	ns	ns	ns	ns	ns
PA time	↓	↓	↓	↓	↓	ns	↓	↑	ns	ns	ns	ns	ns
Steps/day	↓	↓	↓	↓	↓	ns	↓	↑	ns	ns	ns	ns	ns
Breaks in sedentary time	↓	↓	↓	↓	↓	↓	↓	↑	ns	ns	↓	ns	ns
MET mean/day	↓	↓	↓	↓	↓	ns	↓	↑	ns	ns	ns	ns	ns
MET peak/day	↓	↓	↓	↓	↓	ns	↓	↑	ns	ns	ns	ns	ns

For lipid-related outcomes, the possible use of cholesterol-lowering medication (statins) was included the model; and for blood pressure-related outcomes, the possible presence of medical treatment of hypertension was included the model.

*BMI* body mass index, *HOMA-IR* homeostasis model for insulin resistance, *HbA1c* glycated hemoglobin, *HDL* high density lipoprotein, *LDL* low density lipoprotein, *RHR* resting heart rate, *SBP* systolic blood pressure, *DBP* diastolic blood pressure; *ns* non-significant, *LPA* light physical activity, *MVPA* moderate to vigorous physical activity, *PA* physical activity (LPA and MVPA together), *MET* the metabolic equivalent.

The significant positive and negative associations are indicated by upward ↑ and downward ↓ arrows, respectively.

### Models adjusted with BMI

Further adjustment by adding BMI to the statistical analyses had a major impact to some of the results, but not all. See Supplementary Tables S14–S18 for details of the BMI-adjusted models. All the associations between breaks in sedentary time and metabolic markers turned non-significant, when BMI was added to the model. However, fasting insulin and HOMA-IR associated persistently negatively with PA, daily steps and MET mean and peak values; and positively with SB percentage, when adjusted for age, sex and BMI. Associations of plasma triglycerides and HDL-cholesterol with SB percentage, PA, steps and MET mean values also remained significant, when adjusted for age, sex, BMI and cholesterol lowering medication. However, all the associations of plasma glucose, HbA_1c_ and resting heart rate with accelerometer measures turned non-significant when BMI was added to the model. All the BMI-adjusted associations are summarized in Table 8.

**Table 8:** Summary table, associations between accelerometer measures and metabolic outcomes derived from clinical investigation and fasting blood samples measured by linear models *with age, sex and BMI included in the analyses*.

	Insulin	HOMA-IR	Glucose	HbA_1c_	Triglycerides	HDL	LDL	Cholesterol	RHR	SBP	DBP
Sedentary %	↑	↑	ns	ns	↑	↓	ns	ns	ns	ns	ns
Sedentary time	ns	ns	ns	ns	ns	↓	ns	ns	ns	ns	ns
Standing %	ns	ns	ns	ns	ns	ns	ns	ns	ns	ns	ns
Standing time	ns	ns	ns	ns	ns	ns	ns	ns	ns	ns	ns
LPA %	↓	↓	ns	ns	↓	↑	ns	ns	ns	ns	ns
LPA time	↓	↓	ns	ns	↓	↑	ns	ns	ns	ns	ns
MVPA %	↓	↓	ns	ns	ns	ns	ns	ns	ns	ns	ns
MVPA time	↓	↓	ns	ns	ns	↑	ns	ns	ns	ns	ns
PA %	↓	↓	ns	ns	↓	↑	ns	ns	ns	ns	ns
PA time	↓	↓	ns	ns	↓	↑	ns	ns	ns	ns	ns
Steps/day	↓	↓	ns	ns	ns	↑	ns	ns	ns	ns	ns
Breaks in sedentary time	ns	ns	ns	ns	ns	ns	ns	ns	ns	ns	ns
MET mean/day	↓	↓	ns	ns	↓	↑	ns	ns	ns	ns	ns
MET peak/day	↓	↓	ns	ns	ns	ns	ns	ns	ns	ns	ns

For lipid-related outcomes, the possible use of cholesterol-lowering medication (statins) was included the model; and for blood pressure-related outcomes, the possible presence of medical treatment of hypertension was included the model.

*HOMA-IR* homeostasis model for insulin resistance, *HbA1c* glycated hemoglobin, *HDL* high density lipoprotein, *LDL* low density lipoprotein, *RHR* resting heart rate, *SBP* systolic blood pressure, *DBP* diastolic blood pressure, *ns* non-significant, *LPA* light physical activity, *MVPA* moderate to vigorous physical activity, *PA* physical activity (LPA and MVPA together), *MET* the metabolic equivalent.

The significant positive and negative associations are indicated by upward ↑ and downward ↓ arrows, respectively.

### Models with 1-week accelerometry

When the analyses were repeated with only the first week’s accelerometer data (4–7 valid days), a number of results were different from the original findings calculated from 4 weeks accelerometer data. The significant associations between fasting insulin and standing percentage as well as LPA time and percentage turned non-significant. Likewise, the associations between HOMA-IR and LPA time and percentage, Triglycerides and standing time and percentage, fasting glucose and total PA and MET peak, HbA_1c_ and daily steps, BMI and total PA time and percentage as well as waist circumference and SB time and total PA time and percentage turned non-significant when analyzed with only the first week’s accelerometer data.

In the models further adjusted with BMI, when examining fasting insulin, only the associations between fasting insulin and MVPA and PA time, daily steps and MET mean remained significant. Similarly, when examining HOMA-IR, only the associations between HOMA-IR and MVPA time and percentage, PA time, daily steps and MET mean remained significant. When examining plasma lipids, there were fewer differences between first week’s and 4 weeks’ accelerometry. The associations between HDL and sedentary time as well as triglycerides and MET mean were non-significant when analyzed with only the first week’s accelerometer data.

## Discussion

In this study, we demonstrate an association between higher SB percentage (of total accelerometer wear time) and insulin resistance (HOMA-IR) in overweight working aged people with low self-reported PA levels. Thus the greater proportion of the day the subjects spent in sedentary activities, the more insulin resistant they were. Furthermore, all physical activity, regardless of the intensity, was associated with lower fasting insulin levels and better insulin sensitivity. PA, and light PA in particular, also was associated with more beneficial plasma lipid profile, but we could not find any associations between breaks in sedentary time and metabolic health markers, when BMI was taken into account.

### Sedentary behavior and health markers

In the present study, total SB time was only negatively associated with HDL-cholesterol, whereas SB percentage (of total accelerometer wear time) was positively associated with fasting insulin, insulin resistance, plasma triglycerides and negatively with HDL-cholesterol, when sex, age, BMI and possible medication were taken into account. Calculating SB percentage attenuates the effect of variance in wear time of the accelerometer; therefore, SB percentage can be considered a more accurate estimate of one’s SB than total SB time. In this study, the smaller the SB percentage, the more insulin sensitive the subjects were and the more favorable was their plasma lipid profile. If BMI was not included in the statistical model, SB percentage also was positively associated with fasting plasma glucose. This suggests that sedentariness plays a role in the regulation of plasma glucose levels, but excessive body adiposity might be a stronger stimulus for elevated plasma glucose than sitting per se. Similarly, in previous studies, sedentary status has been associated with HDL and HOMA-IR when controlled for BMI^15,17^. Without including BMI, the findings have been controversial: SB percentage has associated with plasma triglycerides, but not with HDL-cholesterol or glucose^14^; and total SB time with both insulin and glucose plus lipid profile and blood pressure in one study^24^; and with fasting insulin but not with glucose, triglycerides or HDL in another^25^. Comparing normoglycemic, glucose intolerant and subjects with type 2 diabetes, SB time was shorter in the healthy group compared to the group with diabetes, but not compared to glucose intolerant subjects, when BMI was included in the model^4^. Indeed, SB time may be the trigger to poorer outcome when glucose intolerance develops into type 2 diabetes, especially in combination with low cardiorespiratory fitness^16^.

### Standing and health markers

In the present study, standing still was not a strong enough stimulus to associate with insulin sensitivity when controlled for BMI; even if the associations were in line with the effects of physical activity, they did not reach statistical significance. There are only few studies assessing the effects of standing, most likely due to challenges in the measurement. It is possible to identify standing posture by a tri-axial accelerometer on the hip^22^, as also applied in the current study; and the same method has indicated an association between longer standing time and smaller waist circumference in a slightly younger population-based cohort^18^. In this study, we were able to replicate this finding, standing associated strongly with waist circumference. However, the causality of this association is not clear. Measured by accelerometers on trunk and thigh, greater standing percentage was associated with more beneficial plasma triglycerides and HDL-cholesterol^26^. Measured by questionnaires, standing was associated with lower all-cause and CVD mortality only among the most inactive^27^. It seems that standing indeed can improve metabolic health, but the amount of standing becomes clinically significant only, if the level of PA is very low. However, in the short term, timing of standing bouts combined with dietary patterns may be of importance in glucose and insulin homeostasis^28,29^.

### Light physical activity and health markers

LPA has been recently proposed to be effective in counteracting the effects of sitting. In this study, beneficial blood lipid profile was associated more strongly with LPA than more intensive MVPA. Earlier, the superiority of LPA over MVPA has not been obvious^6,14,26,30^. These discrepancies can be at least partly explained with different study populations and methodologies, including possibly different cutoff points for PA categories. However, investigating the whole plasma lipidome can provide a more detailed insight on this topic. In a recent study by Henson et al., an association between LPA and HDL particles was found, whereas VLDL particles associated strongest with MVPA^31^. Additionally, in a compositional model of total PA and SB time predicting plasma triglycerides, the coefficient vector of LPA was the steepest compared to the other behaviors^26^. Therefore, being active at low intensities, or rather staying lightly active throughout the day, can help maintain a more beneficial plasma lipid profile, if the recommended amount of MVPA is not met.

### Moderate to vigorous physical activity, steps and health markers

As could be expected, in the present study, MVPA was associated with better insulin sensitivity and beneficial plasma lipid profile. The associations between metabolic health outcomes and MVPA were in general somewhat stronger than those of SB percentage and health. This is in line with previous studies, that vigorous activity can to some extend overcome the detrimental effects of SB^2,32^. However, total PA and step count were the strongest predictors of insulin sensitivity and beneficial lipid profile. This can indicate that the total daily duration of PA, regardless of how it is accumulated, is actually more relevant than intensity, when metabolic health is considered. Interestingly, total PA, regardless of intensity, has also been associated with reduced risk for premature mortality^10^. In addition, plasma triglycerides and HDL-cholesterol were better predicted by average MET of the day than daily step count in this study. Steps are accumulated most efficiently in MVPA, therefore it is not surprising that the effects of MVPA and steps were very similar.

### Breaks in sedentary time and health markers

Previously, breaks in sedentary time have been associated with beneficial metabolic health markers^18,19^. However, we managed to detect significant associations between insulin sensitivity and breaks in sedentary time only, if BMI was not included as a variable in the statistical model. It is possible, that breaking sedentary time to shorter bouts does have a minor effect on insulin sensitivity, but the stimulus might not be strong enough to exceed the detrimental effect of excessive body adiposity. Similar to our findings, number of sedentary breaks did not associate with glucose metabolism status measured by oral glucose tolerance test in a comparable study population in the Maastricht Study^4^. As BMI and waist circumference associate negatively with breaks in sedentary time, it appears that overweight and obese persons tend to have fewer breaks in their SB during the day.

### Methodological considerations

The optimal way to measure PA and SB with accelerometers has been discussed^33–35^. The thigh has been proposed to be the optimal site to place the accelerometer^34^. However, the method is rather demanding and can cause discomfort and hypersensitization to the subject, especially if worn for longer periods. Therefore, measurements of longer duration can become unfeasible. We aimed at measuring PA and SB with accelerometers for four consecutive weeks. Therefore, the accelerometer was placed on the right hip with elastic belt to enable extended measurement.

Our findings confirm that measured SB is sensitive to the wear time of the accelerometer^36^. Interpretation of the results were different depending on whether SB was calculated as hours or percentage per day. At the same time, interpretation of PA was independent of the calculation method. It can be presumed that non-wear time during waking hours is most likely sedentary time. Therefore, in the future, accelerometry for full 24 h per day would likely produce more robust and reliable outcome measures.

There occurs considerable intra-individual variation in daily PA and SB. Based on earlier findings, at least 14 days of measurement are needed to give accurate and reliable estimates of individual’s true habitual PA and SB with ICC ≥ 0.9^20,37^. In this study, we measured PA and SB for mean 25 (SD 4) days. There are only few studies that have reported results of a longer wear time. Therefore, we state that this is the first study to report reliable estimates of subjects’ PA, standing and SB of a nearly four week’s measurement, which is to the best of our knowledge the longest duration thus far.

When the analyses were repeated with only the first week’s accelerometer results, a number of conclusions were different from the original results calculated from 4 weeks accelerometer data. Especially the conclusions concerning outcomes related to insulin and glucose metabolism would have been critically different if we had used only 1 week’s accelerometry. This is likely because data variability decreases with more collected data points and therefore the probability of detecting significant associations increases by a longer measurement period. Plasma lipids had less variation in this study sample, and concerning lipids nearly the same conclusions could be drawn from one week and four weeks of accelerometry. Therefore we can recommend longer measurement periods in the future, especially if the purpose is to investigate associations between light activities and outcomes related to insulin action.

### Study limitations

It should be recognized, that in addition to PA and SB there are other elements also contributing to metabolic homeostasis. Energy intake and nutrition play crucial roles in maintaining energy balance and staying healthy. However, we focused on evaluating the associations between PA, SB and markers of metabolic health. It was beyond the scope of this study to evaluate the effects of nutrition to these factors. However, we managed to demonstrate associations between SB, PA and markers of metabolic health controlled for BMI, which reflects the long-term energy balance of the subjects.

In this study the accelerometer was worn during waking hours. Therefore, wear time of the accelerometer can have an impact on the results and reduce measured sedentary time, if the accelerometer is for example removed in the evening well before going to bed. In the future, accelerometry for full 24 h per day would likely produce more robust and reliable outcome measures.

### Conclusions

We observed an association between SB and insulin resistance (HOMA-IR) in overweight working aged adults with low self-reported PA levels. Although BMI is strongly related to plasma glucose, insulin and lipid levels, sitting less and moving more seem to associate with lower plasma insulin and insulin resistance regardless of BMI, whereas overweight may have a stronger impact on glucose than either PA or SB have. Furthermore, all PA regardless of intensity associated with lower fasting insulin and better insulin sensitivity. Especially LPA associated with more beneficial plasma lipid profile, but we could not find any associations between breaks in sedentary time and metabolic health markers, when overweight was taken into account. The total daily duration of PA may be more relevant than intensity in maintaining metabolic health in overweight adults, if the current guidelines for PA are not met. Short breaks or bursts of MVPA may not provide sufficient stimuli to preserve metabolic homeostasis with concurrent overweight, if the majority of the day is spent sedentary.

## Supplementary information

Supplementary Information.

## Data Availability

The datasets generated during the current study are available from the corresponding author on reasonable request.
