# Supplementary material for: Both sedentary time and physical activity are associated with cardiometabolic health in overweight adults in a 1 month accelerometer measurement
Source: Sci Rep. 2020 Nov 25;10:20578. doi: 10.1038/s41598-020-77637-3 (PMC7688927; doi:10.1038/s41598-020-77637-3)
Supplement: Supplementary file 1 — Supplementary Information. [file 41598_2020_77637_MOESM1_ESM.pdf]

Supplementary Information

# Both sedentary time and physical activity are associated with cardiometabolic health in overweight adults in a one month accelerometer measurement

Tanja Sjöros[1], Henri Vähä-Ypyä[2], Saara Laine[1], Taru Garthwaite[1], Minna Lahesmaa[1], Sanna M. Laurila[1,3], Aino Latva-Rasku[1], Anna Savolainen[1], Annika Miikkulainen[1], Eliisa Löyttyniemi[4], Harri Sievänen[2], Kari K. Kalliokoski[1], Juhani Knuuti[1], Tommi Vasankari[2] and Ilkka H. A. Heinonen[1,5]

[1]Turku PET Centre, University of Turku and Turku University Hospital, Turku, Finland; [2]The UKK-Institute, Tampere, Finland; [3]Turku Heart Center, Turku University Hospital, Turku, Finland; [4]Department of Biostatistics, University of Turku, Turku, Finland; [5]Rydberg Laboratory of Applied Sciences, University of Halmstad, Halmstad, Sweden.

Supplementary Tables

Supplementary Table S1. Associations between waist circumference and accelerometer measures, least squares effect tests with sex and age included in the model.

	df	F Ratio	t Ratio	*p*	r^2
Sedentary %	1	11.14	3.34	0.0011	0.21
Sedentary time	1	4.75	2.18	0.031	0.18
Standing %	1	9.80	-3.13	0.0021	0.20
Standing time	1	10.63	-3.26	0.0014	0.21
LPA %	1	1.67	-1.29	0.20	0.16
LPA time	1	2.28	-1.51	0.13	0.16
MVPA %	1	8.55	-2.92	0.004	0.20
MVPA time	1	9.81	-3.13	0.0021	0.20
PA %	1	5.34	-2.31	0.022	0.18
PA time	1	6.31	-2.51	0.013	0.18
Steps / day	1	14.90	-3.86	0.0002	0.23
Breaks in sedentary time	1	19.48	-4.41	<.0001	0.25
MET mean / day	1	10.71	-3.27	0.0013	0.21
MET peak / day	1	14.92	-3.86	0.0002	0.23

df, degrees of freedom; r^2, the coefficient of determination; LPA, light physical activity; MVPA, moderate to vigorous physical activity; PA, physical activity (LPA and MVPA together); MET, the metabolic equivalent.

Supplementary Table S2. Full model estimates, least squares effect tests for response variable BMI, with sex, age and an activity measure included in the model.

	df	F-ratio	t-ratio	*p*
sex[male]	1	0.33	-0.57	0.57

age	1	1.99	-1.41	0.16
sedentary %	1	7.20	2.68	0.0082
sex[male]	1	0.02	-0.15	0.88
age	1	1.02	-1.01	0.31
sedentary time	1	2.27	1.51	0.13
sex[male]	1	0.76	-0.87	0.39
age	1	1.83	-1.35	0.18
standing %	1	5.91	-2.43	0.016
sex[male]	1	0.85	-0.92	0.36
age	1	2.01	-1.42	0.16
standing time	1	6.73	-2.59	0.011
sex[male]	1	0.00	0.07	0.94
age	1	0.80	-0.90	0.37
LPA %	1	0.93	-0.97	0.34
sex[male]	1	0.00	0.03	0.97
age	1	0.89	-0.94	0.35
LPA time	1	1.61	-1.27	0.21
sex[male]	1	0.27	0.52	0.60
age	1	2.70	-1.64	0.10
MVPA %	1	6.92	-2.63	0.0095
sex[male]	1	0.26	0.51	0.61
age	1	3.09	-1.76	0.081
MVPA time	1	8.08	-2.84	0.0051
sex[male]	1	0.02	0.14	0.89
age	1	1.37	-1.17	0.24
PA %	1	3.79	-1.95	0.054
sex[male]	1	0.01	0.12	0.91
age	1	1.62	-1.27	0.21
PA time	1	4.88	-2.21	0.029
sex[male]	1	0.28	0.53	0.60
age	1	4.15	-2.04	0.044
steps/day	1	13.81	-3.72	0.0003
sex[male]	1	0.57	-0.75	0.45
age	1	2.84	-1.68	0.094
breaks in sedentary time	1	16.24	-4.03	<.0001

sex[male]	1	0.17	0.41	0.68
age	1	2.95	-1.72	0.088
MET mean/day	1	9.08	-3.01	0.0031
sex[male]	1	0.41	0.64	0.52
age	1	6.52	-2.55	0.012
MET peak/day	1	12.63	-3.55	0.0005

df, degrees of freedom; LPA, light physical activity; MVPA, moderate to vigorous physical activity; PA, physical activity (LPA and MVPA together); MET, the metabolic equivalent.

Supplementary Table S3. Full model estimates, least squares effect tests for response variable waist circumference, with sex, age and an activity measure included in the model.

	df	F-ratio	t-ratio	*p*
sex[male]	1	14.33	3.79	0.0002
age	1	0.01	-0.07	0.94
sedentary %	1	11.14	3.34	0.0011
sex[male]	1	17.79	4.22	<.0001
age	1	0.16	0.40	0.69
sedentary time	1	4.75	2.18	0.031
sex[male]	1	9.50	3.08	0.0025
age	1	0.00	-0.03	0.98
standing %	1	9.80	-3.13	0.0021
sex[male]	1	9.45	3.07	0.0025
age	1	0.01	-0.09	0.93
standing time	1	10.63	-3.26	0.0014
sex[male]	1	20.97	4.58	<.0001
age	1	0.31	0.55	0.58
LPA %	1	1.67	-1.29	0.20
sex[male]	1	20.85	4.57	<.0001
age	1	0.25	0.50	0.62
LPA time	1	2.28	-1.51	0.13
sex[male]	1	27.47	5.24	<.0001
age	1	0.10	-0.31	0.76
MVPA %	1	8.55	-2.92	0.004
sex[male]	1	27.58	5.25	<.0001
age	1	0.19	-0.43	0.67
MVPA time	1	9.81	-3.13	0.0021

sex[male]	1	22.9	4.79	<.0001
age	1	0.05	0.23	0.82
PA %	1	5.34	-2.31	0.022
sex[male]	1	22.83	4.78	<.0001
age	1	0.02	0.13	0.90
PA time	1	6.31	-2.51	0.013
sex[male]	1	28.53	5.34	<.0001
age	1	0.41	-0.64	0.52
steps/day	1	14.90	-3.86	0.0002
sex[male]	1	15.22	3.90	0.0001
age	1	0.07	-0.26	0.80
breaks in sedentary time	1	19.48	-4.41	<.0001
sex[male]	1	26.70	5.17	<.0001
age	1	0.12	-0.35	0.72
MET mean/day	1	10.71	-3.27	0.0013
sex[male]	1	29.84	5.46	<.0001
age	1	1.92	-1.38	0.17
MET peak/day	1	14.92	-3.86	0.0002

df, degrees of freedom; LPA, light physical activity; MVPA, moderate to vigorous physical activity; PA, physical activity (LPA and MVPA together); MET, the metabolic equivalent.

Supplementary Table S4. Associations between HOMA-IR and accelerometer measures, least squares effect tests with sex and age included in the model.

	df	F Ratio	t Ratio	*p*	r^2
Sedentary %	1	12.03	3.47	0.0007	0.12
Sedentary time	1	3.41	1.85	0.067	0.07
Standing %	1	5.07	-2.25	0.026	0.08
Standing time	1	6.15	-2.48	0.014	0.09
LPA %	1	4.81	-2.19	0.030	0.08
LPA time	1	6.00	-2.45	0.016	0.08
MVPA %	1	13.06	-3.61	0.0004	0.13
MVPA time	1	15.47	-3.93	0.0001	0.14
PA %	1	10.67	-3.27	0.0014	0.11
PA time	1	12.53	-3.54	0.0005	0.12
Steps / day	1	21.41	-4.63	<.0001	0.17
Breaks in sedentary time	1	13.03	-3.61	0.0004	0.13
MET mean / day	1	16.00	-4.00	0.0001	0.14
MET peak / day	1	14.21	-3.77	0.0002	0.13

df, degrees of freedom; r^2, the coefficient of determination; LPA, light physical activity; MVPA, moderate to vigorous physical activity; PA, physical activity (LPA and MVPA together); MET, the metabolic equivalent.

Supplementary Table S5. Full model estimates, least squares effect tests for response variable fasting plasma insulin (log10), with sex, age and an activity measure included in the model.

	df	F-ratio	t-ratio	*p*
sex[male]	1	3.15	1.77	0.078
age	1	2.05	-1.43	0.15
sedentary %	1	11.15	3.34	0.0011
sex[male]	1	4.98	2.23	0.027
age	1	0.91	-0.95	0.34
sedentary time	1	3.25	1.80	0.073
sex[male]	1	2.57	1.60	0.11
age	1	1.43	-1.20	0.23
standing %	1	4.24	-2.06	0.041
sex[male]	1	2.45	1.56	0.12
age	1	1.58	-1.26	0.21
standing time	1	5.05	-2.25	0.026
sex[male]	1	6.05	2.46	0.015
age	1	0.58	-0.76	0.45
LPA %	1	5.10	-2.26	0.026
sex[male]	1	6.08	2.47	0.015
age	1	0.73	-0.85	0.40
LPA time	1	6.13	-2.48	0.015
sex[male]	1	10.41	3.23	0.0016
age	1	3.27	-1.81	0.073
MVPA %	1	12.16	-3.49	0.0007
sex[male]	1	10.54	3.25	0.0015
age	1	3.79	-1.95	0.054
MVPA time	1	14.03	-3.75	0.0003
sex[male]	1	7.47	2.73	0.0071
age	1	1.52	-1.23	0.22
PA %	1	10.56	-3.25	0.0015
sex[male]	1	7.52	2.74	0.0069
age	1	1.87	-1.37	0.17
PA time	1	12.05	-3.47	0.0007

sex[male]	1	10.68	3.27	0.0014
age	1	4.69	-2.17	0.030
steps/day	1	19.41	-4.41	<.0001
sex[male]	1	3.91	1.98	0.050
age	1	2.15	-1.46	0.15
breaks in sedentary time	1	12.70	-3.56	0.0005
sex[male]	1	9.73	3.12	0.0022
age	1	3.54	-1.88	0.062
MET mean/day	1	15.43	-3.93	0.0001
sex[male]	1	10.34	3.22	0.0016
age	1	6.58	-2.57	0.011
MET peak/day	1	13.53	-3.68	0.0003

df, degrees of freedom; LPA, light physical activity; MVPA, moderate to vigorous physical activity; PA, physical activity (LPA and MVPA together); MET, the metabolic equivalent.

Supplementary Table S6. Full model estimates, least squares effect tests for response variable HOMA-IR (log10), with sex, age and an activity measure included in the model.

	df	F-ratio	t-ratio	*p*
sex[male]	1	2.30	1.52	0.13
age	1	1.65	-1.28	0.20
sedentary %	1	12.03	3.47	0.0007
sex[male]	1	3.99	2.00	0.048
age	1	0.62	-0.79	0.43
sedentary time	1	3.41	1.85	0.067
sex[male]	1	1.74	1.32	0.19
age	1	1.13	-1.06	0.29
standing %	1	5.07	-2.25	0.026
sex[male]	1	1.60	1.26	0.21
age	1	1.28	-1.13	0.26
standing time	1	6.15	-2.48	0.014
sex[male]	1	5.00	2.24	0.027
age	1	0.35	-0.59	0.55
LPA %	1	4.81	-2.19	0.030
sex[male]	1	5.00	2.24	0.027
age	1	0.46	-0.68	0.50
LPA time	1	6.00	-2.45	0.016

sex[male]	1	9.08	3.01	0.0031
age	1	2.81	-1.68	0.096
MVPA %	1	13.06	-3.61	0.0004
sex[male]	1	9.25	3.04	0.0028
age	1	3.37	-1.84	0.068
MVPA time	1	15.47	-3.93	0.0001
sex[male]	1	6.24	2.50	0.014
age	1	1.13	-1.06	0.29
PA %	1	10.67	-3.27	0.0014
sex[male]	1	6.29	2.51	0.013
age	1	1.45	-1.21	0.23
PA time	1	12.53	-3.54	0.0005
sex[male]	1	9.37	3.06	0.0026
age	1	4.26	-2.06	0.041
steps/day	1	21.41	-4.63	<.0001
sex[male]	1	3.02	1.74	0.085
age	1	1.69	-1.30	0.20
breaks in sedentary time	1	13.03	-3.61	0.0004
sex[male]	1	8.34	2.89	0.0045
age	1	2.99	-1.73	0.086
MET mean/day	1	16.00	-4.00	0.0001
sex[male]	1	8.96	2.99	0.0033
age	1	6.04	-2.46	0.015
MET peak/day	1	14.21	-3.77	0.0002

df, degrees of freedom; LPA, light physical activity; MVPA, moderate to vigorous physical activity; PA, physical activity (LPA and MVPA together); MET, the metabolic equivalent.

Supplementary Table S7. Full model estimates, least squares effect tests for response variable fasting plasma glucose, with sex, age and an activity measure included in the model.

	df	F-ratio	t-ratio	*p*
sex[male]	1	0.20	-0.45	0.66
age	1	0.04	-0.20	0.84
sedentary %	1	5.82	2.41	0.017
sex[male]	1	0.01	-0.10	0.92
age	1	0.02	0.13	0.89

sedentary time	1	1.87	1.37	0.17
sex[male]	1	0.47	-0.69	0.49
age	1	0.02	-0.15	0.88
standing %	1	4.38	-2.09	0.038
sex[male]	1	0.58	-0.76	0.45
age	1	0.05	-0.21	0.83
standing time	1	5.37	-2.32	0.022
sex[male]	1	0.01	0.11	0.91
age	1	0.06	0.25	0.80
LPA %	1	0.92	-0.96	0.34
sex[male]	1	0.01	0.09	0.93
age	1	0.05	0.21	0.83
LPA time	1	1.41	-1.19	0.24
sex[male]	1	0.27	0.52	0.60
age	1	0.21	-0.46	0.65
MVPA %	1	5.79	-2.41	0.018
sex[male]	1	0.29	0.54	0.59
age	1	0.36	-0.60	0.55
MVPA time	1	7.45	-2.73	0.0072
sex[male]	1	0.04	0.19	0.85
age	1	0.00	0.01	1.00
PA %	1	3.33	-1.82	0.070
sex[male]	1	0.03	0.18	0.85
age	1	0.01	-0.09	0.93
PA time	1	4.41	-2.10	0.038
sex[male]	1	0.25	0.50	0.61
age	1	0.50	-0.71	0.48
steps/day	1	9.54	-3.09	0.0024
sex[male]	1	0.07	-0.26	0.79
age	1	0.03	-0.16	0.87
breaks in sedentary time	1	4.98	-2.23	0.027
sex[male]	1	0.15	0.39	0.69
age	1	0.17	-0.41	0.68
MET mean/day	1	5.54	-2.35	0.020

sex[male]	1	0.21	0.46	0.65
age	1	0.73	-0.85	0.40
MET peak/day	1	4.54	-2.13	0.035

df, degrees of freedom; LPA, light physical activity; MVPA, moderate to vigorous physical activity; PA, physical activity (LPA and MVPA together); MET, the metabolic equivalent.

Supplementary Table S8. Associations between HbA_{1c} and accelerometer measures, least squares effect tests with sex and age included in the model.

	df	F Ratio	t Ratio	*p*	r^2
Sedentary %	1	1.36	1.16	0.25	0.06
Sedentary time	1	0.62	0.79	0.43	0.05
Standing %	1	0.37	-0.61	0.54	0.05
Standing time	1	0.42	-0.64	0.52	0.05
LPA %	1	0.42	-0.65	0.52	0.05
LPA time	1	0.59	-0.77	0.44	0.05
MVPA %	1	2.70	-1.64	0.10	0.07
MVPA time	1	3.26	-1.81	0.073	0.07
PA %	1	1.55	-1.25	0.21	0.06
PA time	1	1.90	-1.38	0.17	0.06
Steps / day	1	3.44	-1.85	0.066	0.07
Breaks in sedentary time	1	4.73	-2.17	0.031	0.08
MET mean / day	1	3.03	-1.74	0.084	0.07
MET peak / day	1	3.51	-1.87	0.063	0.07

df, degrees of freedom; r^2, the coefficient of determination; LPA, light physical activity; MVPA, moderate to vigorous physical activity; PA, physical activity (LPA and MVPA together); MET, the metabolic equivalent.

Supplementary Table S9. Full model estimates, least squares effect tests for response variable fasting plasma triglycerides (log10), with sex, age and an activity measure included in the model.

	df	F-ratio	t-ratio	*p*
sex[male]	1	0.08	0.29	0.77
age	1	0.17	0.41	0.68
CL medication [0]	1	0.04	0.19	0.85
sedentary %	1	11.05	3.32	0.0011
sex[male]	1	0.71	0.84	0.40
age	1	0.80	0.89	0.37
CL medication [0]	1	0.10	0.32	0.75
sedentary time	1	2.30	1.52	0.13
sex[male]	1	0.05	0.22	0.83
age	1	0.38	0.62	0.54
CL medication [0]	1	0.17	0.42	0.68

standing %	1	4.69	-2.16	0.032
sex[male]	1	0.03	0.18	0.85
age	1	0.33	0.57	0.57
CL medication [0]	1	0.23	0.48	0.64
standing time	1	5.48	-2.34	0.021
sex[male]	1	0.81	0.90	0.37
age	1	1.22	1.11	0.27
CL medication [0]	1	0.05	0.22	0.83
LPA %	1	7.83	-2.80	0.0059
sex[male]	1	0.84	0.92	0.36
age	1	1.04	1.02	0.31
CL medication [0]	1	0.10	0.32	0.75
LPA time	1	9.20	-3.03	0.0029
sex[male]	1	2.42	1.56	0.12
age	1	0.08	0.28	0.78
CL medication [0]	1	0.12	0.35	0.73
MVPA %	1	6.11	-2.47	0.015
sex[male]	1	2.48	1.57	0.12
age	1	0.03	0.18	0.86
CL medication [0]	1	0.18	0.43	0.67
MVPA time	1	7.23	-2.69	0.0080
sex[male]	1	1.41	1.19	0.24
age	1	0.40	0.63	0.53
CL medication [0]	1	0.03	0.19	0.85
PA %	1	9.73	-3.12	0.0022
sex[male]	1	1.45	1.20	0.23
age	1	0.27	0.52	0.60
CL medication [0]	1	0.10	0.32	0.75
PA time	1	11.17	-3.34	0.0011
sex[male]	1	2.28	1.51	0.13
age	1	0.03	0.17	0.87
CL medication [0]	1	0.15	0.39	0.70
steps/day	1	7.34	-2.71	0.0076
sex[male]	1	0.45	0.67	0.50
age	1	0.32	0.57	0.57
CL medication [0]	1	0.3	0.55	0.59
breaks in sedentary time	1	7.25	-2.69	0.008

sex[male]	1	2.16	1.47	0.14
age	1	0.03	0.18	0.86
CL medication [0]	1	0.06	0.25	0.80
MET mean/day	1	8.96	-2.99	0.0033
sex[male]	1	2.18	1.48	0.14
age	1	0.01	-0.08	0.93
CL medication [0]	1	0.22	0.47	0.64
MET peak/day	1	3.98	-2.00	0.048

df, degrees of freedom; CL medication [0], no cholesterol lowering medication; LPA, light physical activity; MVPA, moderate to vigorous physical activity; PA, physical activity (LPA and MVPA together); MET, the metabolic equivalent.

Supplementary Table S10. Full model estimates, least squares effect tests for response variable plasma HDL-cholesterol, with sex, age, cholesterol lowering medication and an activity measure included in the model.

	df	F-ratio	t-ratio	*p*
sex[male]	1	20.15	-4.49	<.0001
age	1	6.47	2.54	0.010
CL medication [0]	1	1.24	-1.11	0.27
sedentary %	1	9.60	-3.10	0.0024
sex[male]	1	22.57	-4.75	<.0001
age	1	4.65	2.16	0.033
CL medication [0]	1	1.19	-1.09	0.28
sedentary time	1	5.92	-2.43	0.016
sex[male]	1	18.64	-4.32	<.0001
age	1	4.76	2.18	0.031
CL medication [0]	1	1.79	-1.34	0.18
standing %	1	2.10	1.45	0.15
sex[male]	1	18.49	-4.3	<.0001
age	1	4.97	2.23	0.028
CL medication [0]	1	1.90	-1.38	0.17
standing time	1	2.65	1.63	0.11
sex[male]	1	26.49	-5.15	<.0001
age	1	3.7	1.92	0.057
CL medication [0]	1	1.17	-1.08	0.28
LPA %	1	9.93	3.15	0.0020
sex[male]	1	27.03	-5.20	<.0001
age	1	4.12	2.03	0.044

CL medication [0]	1	1.49	-1.22	0.22
LPA time	1	10.53	3.24	0.0015
sex[male]	1	34.18	-5.85	<.0001
age	1	7.11	2.67	0.0086
CL medication [0]	1	1.53	-1.24	0.22
MVPA %	1	7.12	2.67	0.0085
sex[male]	1	34.48	-5.87	<.0001
age	1	7.55	2.75	0.0068
CL medication [0]	1	1.76	-1.33	0.19
MVPA time	1	8.03	2.83	0.0053
sex[male]	1	30.7	-5.54	<.0001
age	1	5.99	2.45	0.016
CL medication [0]	1	1.13	-1.06	0.29
PA %	1	11.98	3.46	0.0007
sex[male]	1	31.04	-5.57	<.0001
age	1	6.47	2.54	0.012
CL medication [0]	1	1.49	-1.22	0.22
PA time	1	12.67	3.56	0.0005
sex[male]	1	34.17	-5.85	<.0001
age	1	7.93	2.82	0.0056
CL medication [0]	1	1.65	-1.28	0.20
steps/day	1	9.05	3.01	0.0031
sex[male]	1	24.00	-4.90	<.0001
age	1	5.49	2.34	0.021
CL medication [0]	1	2.08	-1.44	0.15
breaks in sedentary time	1	5.56	2.36	0.020
sex[male]	1	34.31	-5.86	<.0001
age	1	8.28	2.88	0.0047
CL medication [0]	1	1.26	-1.12	0.26
MET mean/day	1	11.76	3.43	0.0008
sex[male]	1	32.59	-5.71	<.0001
age	1	7.40	2.72	0.0074
CL medication [0]	1	1.86	-1.36	0.18
MET peak/day	1	4.19	2.05	0.043

df, degrees of freedom; CL medication [0], no cholesterol lowering medication; LPA, light physical activity; MVPA, moderate to vigorous physical activity; PA, physical activity (LPA and MVPA together); MET, the metabolic equivalent.

Supplementary Table S11. Associations between total plasma cholesterol and accelerometer measures, least squares effect tests with sex, age and cholesterol lowering medication included in the model.

	df	F Ratio	t Ratio	*p*	r^2
Sedentary %	1	0.07	-0.26	0.80	0.11
Sedentary time	1	0.51	-0.71	0.48	0.11
Standing %	1	0.11	0.33	0.74	0.11
Standing time	1	0.17	0.42	0.68	0.11
LPA %	1	0.38	0.62	0.54	0.11
LPA time	1	0.32	0.56	0.57	0.11
MVPA %	1	0.38	-0.61	0.54	0.11
MVPA time	1	0.35	-0.59	0.56	0.11
PA %	1	0.01	0.11	0.92	0.11
PA time	1	0.01	0.09	0.93	0.11
Steps / day	1	0.04	-0.2	0.84	0.11
Breaks in sedentary time	1	2.09	1.45	0.15	0.12
MET mean / day	1	0.06	-0.24	0.81	0.11
MET peak / day	1	0.75	-0.87	0.39	0.12

df, degrees of freedom; r^2, the coefficient of determination; LPA, light physical activity; MVPA, moderate to vigorous physical activity; PA, physical activity (LPA and MVPA together); MET, the metabolic equivalent.

Supplementary Table S12. Associations between total plasma LDL-cholesterol and accelerometer measures, least squares effect tests with sex, age and cholesterol lowering medication included in the model.

	df	F Ratio	t Ratio	*p*	r^2
Sedentary %	1	0.10	0.31	0.75	0.09
Sedentary time	1	0.00	0.05	0.96	0.09
Standing %	1	0.00	0.02	0.98	0.09
Standing time	1	0.01	0.11	0.91	0.09
LPA %	1	0.09	0.30	0.76	0.09
LPA time	1	0.06	0.25	0.81	0.09
MVPA %	1	2.02	-1.42	0.16	0.11
MVPA time	1	2.04	-1.43	0.16	0.11
PA %	1	0.27	-0.52	0.60	0.09
PA time	1	0.27	-0.52	0.60	0.09
Steps / day	1	1.16	-1.08	0.28	0.10
Breaks in sedentary time	1	1.90	1.38	0.17	0.10
MET mean / day	1	1.09	-1.04	0.30	0.10
MET peak / day	1	2.07	-1.44	0.15	0.11

df, degrees of freedom; r^2, the coefficient of determination; LPA, light physical activity; MVPA, moderate to vigorous physical activity; PA, physical activity (LPA and MVPA together); MET, the metabolic equivalent.

Supplementary Table S13. Full model estimates, least squares effect tests for response variable total plasma cholesterol with sex, age, cholesterol lowering medication and an activity measure included in the model.

	df	F-ratio	t-ratio	*p*
sex[male]	1	5.43	-2.33	0.021
age	1	5.04	2.25	0.026
CL medication [0]	1	6.58	2.57	0.011
sedentary %	1	0.07	-0.26	0.80
sex[male]	1	5.07	-2.25	0.026
age	1	5.22	2.28	0.024
CL medication [0]	1	6.92	2.63	0.010
sedentary time	1	0.51	-0.71	0.48
sex[male]	1	4.52	-2.13	0.035
age	1	5.10	2.26	0.026
CL medication [0]	1	6.59	2.57	0.011
standing %	1	0.11	0.33	0.74
sex[male]	1	4.43	-2.11	0.037
age	1	5.18	2.28	0.024
CL medication [0]	1	6.58	2.56	0.011
standing time	1	0.17	0.42	0.68
sex[male]	1	5.80	-2.41	0.017
age	1	4.93	2.22	0.028
CL medication [0]	1	6.82	2.61	0.010
LPA %	1	0.38	0.62	0.54
sex[male]	1	5.89	-2.43	0.017
age	1	5.02	2.24	0.027
CL medication [0]	1	6.71	2.59	0.011
LPA time	1	0.32	0.56	0.57
sex[male]	1	5.94	-2.44	0.016
age	1	3.90	1.97	0.050
CL medication [0]	1	6.25	2.50	0.014
MVPA %	1	0.38	-0.61	0.54
sex[male]	1	5.98	-2.44	0.016
age	1	3.86	1.96	0.052
CL medication [0]	1	6.39	2.53	0.013
MVPA time	1	0.35	-0.59	0.56
sex[male]	1	6.30	-2.51	0.013
age	1	4.98	2.23	0.027

CL medication [0]	1	6.49	2.55	0.012
PA %	1	0.01	0.11	0.92
sex[male]	1	6.31	-2.51	0.013
age	1	4.94	2.22	0.028
CL medication [0]	1	6.51	2.55	0.012
PA time	1	0.01	0.09	0.93
sex[male]	1	6.21	-2.49	0.014
age	1	4.30	2.07	0.040
CL medication [0]	1	6.44	2.54	0.012
steps/day	1	0.04	-0.20	0.84
sex[male]	1	4.63	-2.15	0.033
age	1	6.04	2.46	0.015
CL medication [0]	1	6.61	2.57	0.011
breaks in sedentary time	1	2.09	1.45	0.15
sex[male]	1	6.25	-2.50	0.014
age	1	4.36	2.09	0.039
CL medication [0]	1	6.33	2.52	0.013
MET mean/day	1	0.06	-0.24	0.81
sex[male]	1	5.88	-2.42	0.017
age	1	2.37	1.54	0.13
CL medication [0]	1	6.39	2.53	0.013
MET peak/day	1	0.75	-0.87	0.39

df, degrees of freedom; CL medication[0], no cholesterol lowering medication; LPA, light physical activity; MVPA, moderate to vigorous physical activity; PA, physical activity (LPA and MVPA together); MET, the metabolic equivalent.

Supplementary Table S14. Full model estimates, least squares effect tests for response variable fasting plasma insulin (log10), with sex, age, BMI and an activity measure included in the model.

	df	F-ratio	t-ratio	*p*
sex[male]	1	6.02	2.45	0.02
age	1	0.64	-0.80	0.42
BMI	1	59.02	7.68	<.0001
sedentary %	1	4.82	2.20	0.030
sex[male]	1	7.78	2.79	0.006
age	1	0.21	-0.46	0.64
BMI	1	64.94	8.06	<.0001
sedentary time	1	1.38	1.17	0.24

sex[male]	1	6.11	2.47	0.01
age	1	0.29	-0.54	0.59
BMI	1	62.57	7.91	<.0001
standing %	1	0.73	-0.86	0.39
sex[male]	1	6.01	2.45	0.016
age	1	0.33	-0.57	0.57
BMI	1	61.75	7.86	<.0001
standing time	1	0.92	-0.96	0.34
sex[male]	1	8.49	2.91	0.0042
age	1	0.10	-0.31	0.76
BMI	1	66.51	8.16	<.0001
LPA %	1	4.28	-2.07	0.041
sex[male]	1	8.60	2.93	0.0039
age	1	0.16	-0.40	0.69
BMI	1	65.32	8.08	<.0001
LPA time	1	4.45	-2.11	0.037
sex[male]	1	12.18	3.49	0.001
age	1	1.18	-1.09	0.279
BMI	1	59.10	7.69	<.0001
MVPA %	1	5.77	-2.40	0.018
sex[male]	1	12.29	3.51	0.0006
age	1	1.41	-1.19	0.24
BMI	1	57.71	7.60	<.0001
MVPA time	1	6.59	-2.57	0.011
sex[male]	1	10.01	3.16	0.0019
age	1	0.51	-0.71	0.48
BMI	1	62.39	7.90	<.0001
PA %	1	6.67	-2.58	0.011
sex[male]	1	10.05	3.17	0.0019
age	1	0.66	-0.81	0.4189
BMI	1	60.92	7.80	<.0001
PA time	1	7.06	-2.66	0.0088
sex[male]	1	12.16	3.49	0.0007
age	1	1.64	-1.28	0.20
BMI	1	52.43	7.24	<.0001
steps/day	1	7.64	-2.76	0.0065

sex[male]	1	7.48	2.73	0.0071
age	1	0.49	-0.70	0.49
BMI	1	53.85	7.34	<.0001
breaks in sedentary time	1	2.52	-1.59	0.11
sex[male]	1	11.68	3.42	0.0008
age	1	1.29	-1.14	0.26
BMI	1	56.64	7.53	<.0001
MET mean/day	1	7.12	-2.67	0.0085
sex[male]	1	11.76	3.43	0.0008
age	1	1.93	-1.39	0.17
BMI	1	56.40	7.51	<.0001
MET peak/day	1	4.34	-2.08	0.039

df, degrees of freedom; LPA, light physical activity; MVPA, moderate to vigorous physical activity; PA, physical activity (LPA and MVPA together); MET, the metabolic equivalent.

Supplementary Table S15. Full model estimates, least squares effect tests for response variable fasting plasma glucose with sex, age, BMI and an activity measure included in the model.

	df	F-ratio	t-ratio	*p*
sex[male]	1	0.09	-0.29	0.77
age	1	0.07	0.26	0.80
BMI	1	16.21	4.03	<.0001
sedentary %	1	2.55	1.60	0.11
sex[male]	1	0.00	-0.06	0.95
age	1	0.26	0.51	0.61
BMI	1	18.59	4.31	<.0001
sedentary time	1	0.83	0.91	0.36
sex[male]	1	0.19	-0.44	0.66
age	1	0.10	0.31	0.76
BMI	1	16.93	4.11	<.0001
standing %	1	1.82	-1.35	0.18
sex[male]	1	0.25	-0.50	0.62
age	1	0.06	0.25	0.80
BMI	1	16.44	4.05	<.0001
standing time	1	2.32	-1.52	0.13
sex[male]	1	0.01	0.08	0.94
age	1	0.35	0.59	0.55
BMI	1	19.21	4.38	<.0001
LPA %	1	0.42	-0.65	0.52

sex[male]	1	0.01	0.07	0.94
age	1	0.32	0.57	0.57
BMI	1	18.87	4.34	<.0001
LPA time	1	0.62	-0.79	0.43
sex[male]	1	0.13	0.36	0.72
age	1	0.00	0.07	0.95
BMI	1	16.3	4.04	<.0001
MVPA %	1	2.62	-1.62	0.11
sex[male]	1	0.15	0.39	0.70
age	1	0.00	-0.05	0.96
BMI	1	15.55	3.94	0.0001
MVPA time	1	3.53	-1.88	0.063
sex[male]	1	0.02	0.14	0.89
age	1	0.17	0.41	0.68
BMI	1	17.76	4.21	<.0001
PA %	1	1.51	-1.23	0.22
sex[male]	1	0.02	0.13	0.89
age	1	0.11	0.34	0.74
BMI	1	17.15	4.14	<.0001
PA time	1	2.03	-1.42	0.16
sex[male]	1	0.12	0.35	0.72
age	1	0.01	-0.10	0.92
BMI	1	13.79	3.71	0.0003
steps/day	1	3.89	-1.97	0.051
sex[male]	1	0.00	-0.06	0.96
age	1	0.13	0.37	0.71
BMI	1	15.29	3.91	0.0001
breaks in sedentary time	1	0.90	-0.95	0.34
sex[male]	1	0.07	0.27	0.79
age	1	0.02	0.14	0.89
BMI	1	15.94	3.99	0.0001
MET mean/day	1	2.03	-1.43	0.16
sex[male]	1	0.08	0.29	0.77
age	1	0.00	-0.03	0.98
BMI	1	16.25	4.03	<.0001
MET peak/day	1	1.07	-1.03	0.30

df, degrees of freedom; LPA, light physical activity; MVPA, moderate to vigorous physical activity; MET, the metabolic equivalent.

Supplementary Table S16. Full model estimates, least squares effect tests for response variable plasma triglycerides (log10), with sex, age, BMI, cholesterol lowering medication and an activity measure included in the model.

	df	F-ratio	t-ratio	*p*
sex[male]	1	0.24	0.49	0.62
age	1	0.72	0.85	0.40
BMI	1	10.06	3.17	0.0019
CL medication [0]	1	0.33	0.57	0.57
sedentary %	1	6.77	2.60	0.010
sex[male]	1	0.96	0.98	0.33
age	1	1.67	1.29	0.20
BMI	1	13.10	3.62	0.0004
CL medication [0]	1	0.54	0.74	0.46
sedentary time	1	1.14	1.07	0.29
sex[male]	1	0.26	0.51	0.61
age	1	1.15	1.07	0.28
BMI	1	11.74	3.43	0.0008
CL medication [0]	1	0.63	0.79	0.43
standing %	1	2.26	-1.50	0.14
sex[male]	1	0.23	0.48	0.63
age	1	1.06	1.03	0.30
BMI	1	11.41	3.38	0.001
CL medication [0]	1	0.69	0.83	0.41
standing time	1	2.68	-1.64	0.10
sex[male]	1	0.91	0.95	0.34
age	1	2.16	1.47	0.14
BMI	1	13.11	3.62	0.0004
CL medication [0]	1	0.36	0.60	0.55
LPA %	1	6.57	-2.56	0.012
sex[male]	1	0.95	0.97	0.33
age	1	1.91	1.38	0.17
BMI	1	12.60	3.55	0.0005
CL medication [0]	1	0.48	0.69	0.49
LPA time	1	7.45	-2.73	0.0072
sex[male]	1	2.24	1.50	0.14

age	1	0.63	0.80	0.43
BMI	1	11.18	3.34	0.0011
CL medication [0]	1	0.54	0.74	0.46
MVPA %	1	3.06	-1.75	0.083
sex[male]	1	2.29	1.51	0.13
age	1	0.50	0.71	0.48
BMI	1	10.71	3.27	0.0014
CL medication [0]	1	0.62	0.79	0.43
MVPA time	1	3.72	-1.93	0.056
sex[male]	1	1.49	1.22	0.22
age	1	1.06	1.03	0.30
BMI	1	11.37	3.37	0.0010
CL medication [0]	1	0.33	0.58	0.57
PA %	1	6.74	-2.60	0.010
sex[male]	1	1.53	1.24	0.22
age	1	0.87	0.93	0.35
BMI	1	10.84	3.29	0.0013
CL medication [0]	1	0.46	0.68	0.50
PA time	1	7.66	-2.77	0.0064
sex[male]	1	2.10	1.45	0.15
age	1	0.55	0.74	0.46
BMI	1	9.75	3.12	0.0022
CL medication [0]	1	0.58	0.76	0.45
steps / day	1	2.91	-1.71	0.090
sex[male]	1	0.80	0.90	0.37
age	1	1.09	1.04	0.30
BMI	1	9.54	3.09	0.0024
CL medication [0]	1	0.74	0.86	0.39
breaks in sedentary time	1	2.62	-1.62	0.11
sex[male]	1	2.08	1.44	0.15
age	1	0.48	0.69	0.49
BMI	1	10.02	3.17	0.0019
CL medication [0]	1	0.41	0.64	0.52
MET mean/day	1	4.73	-2.18	0.031
sex[male]	1	2.02	1.42	0.16
age	1	0.38	0.61	0.54
BMI	1	11.25	3.35	0.0010
CL medication [0]	1	0.67	0.82	0.41

MET peak/day	1	1.33	-1.15	0.25

df, degrees of freedom; CL medication [0], no cholesterol lowering medication; LPA, light physical activity; MVPA, moderate to vigorous physical activity; PA, physical activity (LPA and MVPA together); MET, the metabolic equivalent.

Supplementary Table S17. Full model estimates, least squares effect tests for response variable plasma HDL-cholesterol, with sex, age, BMI, cholesterol lowering medication and an activity measure included in the model.

	df	F-ratio	t-ratio	*p*
sex[male]	1	23.32	-4.83	<.0001
age	1	4.71	2.17	0.032
BMI	1	10.51	-3.24	0.0015
CL medication [0]	1	2.35	-1.53	0.13
sedentary %	1	5.60	-2.37	0.019
sex[male]	1	25.43	-5.04	<.0001
age	1	3.51	1.87	0.063
BMI	1	12.68	-3.56	0.0005
CL medication [0]	1	2.32	-1.52	0.13
sedentary time	1	4.08	-2.02	0.046
sex[male]	1	22.8	-4.77	<.0001
age	1	3.25	1.80	0.074
BMI	1	12.89	-3.59	0.0005
CL medication [0]	1	3.12	-1.77	0.08
standing %	1	0.56	0.75	0.45
sex[male]	1	22.69	-4.76	<.0001
age	1	3.37	1.83	0.069
BMI	1	12.58	-3.55	0.0005
CL medication [0]	1	3.19	-1.79	0.076
standing time	1	0.79	0.89	0.377
sex[male]	1	29.01	-5.39	<.0001
age	1	2.81	1.68	0.096
BMI	1	13.30	-3.65	0.0004
CL medication [0]	1	2.26	-1.50	0.14
LPA %	1	8.61	2.94	0.0039
sex[male]	1	29.56	-5.44	<.0001
age	1	3.16	1.78	0.078
BMI	1	12.77	-3.57	0.0005
CL medication [0]	1	2.65	-1.63	0.11
LPA time	1	8.68	2.95	0.0038

sex[male]	1	35.31	-5.94	<.0001
age	1	4.95	2.22	0.028
BMI	1	11.15	-3.34	0.0011
CL medication [0]	1	2.72	-1.65	0.1
MVPA %	1	3.83	1.96	0.052
sex[male]	1	35.52	-5.96	<.0001
age	1	5.22	2.28	0.024
BMI	1	10.74	-3.28	0.0013
CL medication [0]	1	2.93	-1.71	0.089
MVPA time	1	4.3	2.07	0.04
sex[male]	1	32.92	-5.74	<.0001
age	1	4.59	2.14	0.034
BMI	1	11.35	-3.37	0.001
CL medication [0]	1	2.18	-1.48	0.14
PA %	1	8.74	2.96	0.0037
sex[male]	1	33.18	-5.76	<.0001
age	1	4.92	2.22	0.028
BMI	1	10.89	-3.30	0.0012
CL medication [0]	1	2.60	-1.61	0.11
PA time	1	8.93	2.99	0.0033
sex[male]	1	34.96	-5.91	<.0001
age	1	5.22	2.29	0.024
BMI	1	9.46	-3.08	0.0025
CL medication [0]	1	2.78	-1.67	0.10
steps / day	1	4.05	2.01	0.046
sex[male]	1	27.75	-5.27	<.0001
age	1	3.66	1.91	0.058
BMI	1	10.39	-3.22	0.0016
CL medication [0]	1	3.23	-1.80	0.075
breaks in sedentary time	1	1.58	1.26	0.21
sex[male]	1	35.57	-5.96	<.0001
age	1	5.9	2.43	0.016
BMI	1	9.68	-3.11	0.0023
CL medication [0]	1	2.33	-1.53	0.13
MET mean/day	1	6.87	2.62	0.0098
sex[male]	1	33.52	-5.79	<.0001
age	1	4.04	2.01	0.046

BMI	1	11.56	-3.40	0.0009
CL medication [0]	1	3.05	-1.75	0.083
MET peak/day	1	1.19	1.09	0.28

df, degrees of freedom; CL medication [0], no cholesterol lowering medication; LPA, light physical activity; MVPA, moderate to vigorous physical activity; PA, physical activity (LPA and MVPA together); MET, the metabolic equivalent.

Supplementary Table S18. Full model estimates, least squares effect tests for response variable total plasma cholesterol, with sex, age, BMI, cholesterol lowering medication and an activity measure included in the model.

	df	F-ratio	t-ratio	*p*
sex[male]	1	5.58	-2.36	0.020
age	1	4.54	2.13	0.035
BMI	1	0.45	-0.67	0.50
CL medication [0]	1	6.06	2.46	0.015
sedentary %	1	0.01	-0.10	0.92
sex[male]	1	5.12	-2.26	0.025
age	1	4.86	2.21	0.029
BMI	1	0.39	-0.62	0.54
CL medication [0]	1	6.45	2.54	0.012
sedentary time	1	0.38	-0.62	0.54
sex[male]	1	4.69	-2.17	0.032
age	1	4.63	2.15	0.033
BMI	1	0.43	-0.66	0.51
CL medication [0]	1	6.14	2.48	0.014
standing %	1	0.03	0.19	0.85
sex[male]	1	4.61	-2.15	0.034
age	1	4.70	2.17	0.032
BMI	1	0.41	-0.64	0.53
CL medication [0]	1	6.16	2.48	0.014
standing time	1	0.07	0.27	0.79
sex[male]	1	5.79	-2.41	0.018
age	1	4.61	2.15	0.034
BMI	1	0.43	-0.66	0.51
CL medication [0]	1	6.37	2.52	0.013
LPA %	1	0.31	0.55	0.58
sex[male]	1	5.88	-2.43	0.017
age	1	4.69	2.17	0.032
BMI	1	0.42	-0.65	0.52
CL medication [0]	1	6.28	2.51	0.013

LPA time	1	0.24	0.48	0.63
sex[male]	1	5.79	-2.41	0.018
age	1	3.30	1.82	0.072
BMI	1	0.76	-0.87	0.39
CL medication [0]	1	5.69	2.39	0.018
MVPA %	1	0.62	-0.79	0.43
sex[male]	1	5.81	-2.41	0.017
age	1	3.23	1.80	0.074
BMI	1	0.77	-0.88	0.38
CL medication [0]	1	5.85	2.42	0.017
MVPA time	1	0.61	-0.78	0.44
sex[male]	1	6.27	-2.5	0.014
age	1	4.55	2.13	0.035
BMI	1	0.50	-0.70	0.48
CL medication [0]	1	5.99	2.45	0.016
PA %	1	0.00	-0.02	0.99
sex[male]	1	6.27	-2.5	0.013
age	1	4.49	2.12	0.036
BMI	1	0.50	-0.71	0.48
CL medication [0]	1	6.04	2.46	0.015
PA time	1	0.00	-0.05	0.96
sex[male]	1	6.05	-2.46	0.015
age	1	3.55	1.88	0.062
BMI	1	0.66	-0.81	0.42
CL medication [0]	1	5.87	2.42	0.017
steps / day	1	0.19	-0.44	0.66
sex[male]	1	4.65	-2.16	0.033
age	1	5.68	2.38	0.019
BMI	1	0.07	-0.26	0.79
CL medication [0]	1	6.38	2.53	0.013
breaks in sedentary time	1	1.63	1.28	0.20
sex[male]	1	6.13	-2.48	0.015
age	1	3.72	1.93	0.056
BMI	1	0.64	-0.80	0.42
CL medication [0]	1	5.75	2.40	0.018
MET mean/day	1	0.19	-0.44	0.66
sex[male]	1	5.59	-2.36	0.020
age	1	1.53	1.24	0.22

BMI	1	1.05	-1.03	0.31
CL medication [0]	1	5.68	2.38	0.019
MET peak/day	1	1.56	-1.25	0.21

df, degrees of freedom; CL medication [0], no cholesterol lowering medication; LPA, light physical activity; MVPA, moderate to vigorous physical activity; MET, the metabolic equivalent.

Supplementary Figures

Supplementary Figure S1. The unadjusted associations between fasting plasma insulin (a, e, i, m, q, u), HOMA-IR (b, f, j, n, r, v), glucose (c, g, k, o, s, w), HbA1c (d, h, l, p, t, x) and accelerometer measures, n 142 (40 men). LPA, light physical activity; MVPA, moderate to vigorous physical activity.

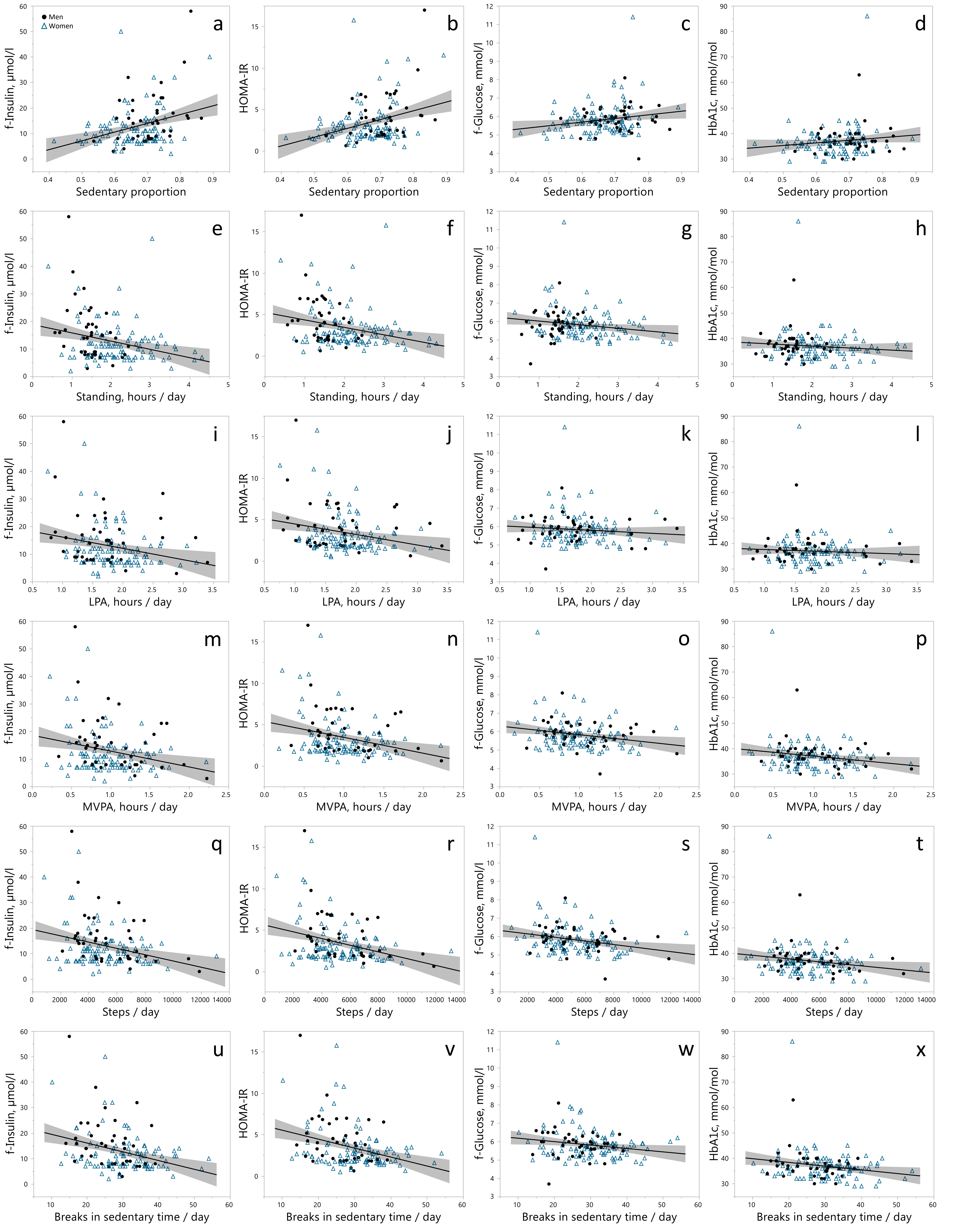

Men
Women
f-Insulin, µmol/l
HOMA-IR
f-Glucose, mmol/l
HbA1c, mmol/mol
Sedentary proportion
Standing, hours / day
LPA, hours / day
MVPA, hours / day
Steps / day
Breaks in sedentary time / day
a
b
c
d
e
f
g
h
i
j
k
l
m
n
o
p
q
r
s
t
u
v
w
x

Supplementary Figure S2. The unadjusted associations between plasma triglycerides (a, e, i, m, q, u), total cholesterol (b, f, j, n, r, v), LDL (c, g, k, o, s, w), HDL (d, h, l, p, t, x) and accelerometer measures, n 142 (40 men). LPA, light physical activity; MVPA, moderate to vigorous physical activity.

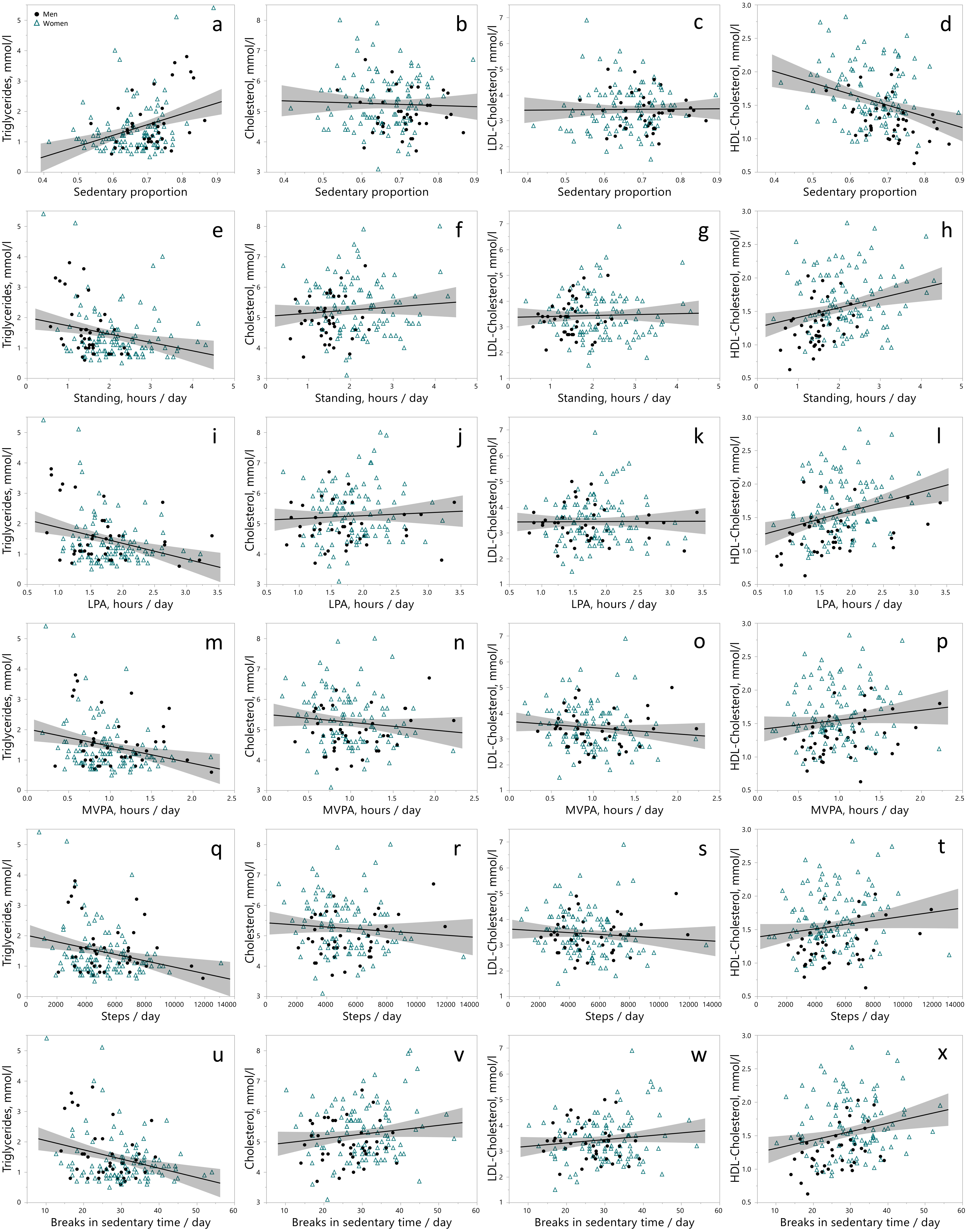
Men
Women
a
b
c
d
e
f
g
h
i
j
k
l
m
n
o
p
q
r
s
t
u
v
w
x
Triglycerides, mmol/l
Cholesterol, mmol/l
LDL-Cholesterol, mmol/l
HDL-Cholesterol, mmol/l
Sedentary proportion
Standing, hours / day
LPA, hours / day
MVPA, hours / day
Steps / day
Breaks in sedentary time / day
